# A representation of a compressed de Bruijn graph for pan-genome analysis that enables search

**DOI:** 10.1186/s13015-016-0083-7

**Published:** 2016-07-18

**Authors:** Timo Beller, Enno Ohlebusch

**Affiliations:** Institute of Theoretical Computer Science, Ulm University, James-Franck-Ring O27/537, 89069 Ulm, Germany

**Keywords:** Compressed de Bruijn graph, Burrows–Wheeler transform, Backward search, Pan-genome analysis

## Abstract

**Background:**

Recently, Marcus et al. (Bioinformatics 30:3476–83, 2014) proposed to use a compressed de Bruijn graph to describe the relationship between the genomes of many individuals/strains of the same or closely related species. They devised an $O(n\log g)$ time algorithm called splitMEM that constructs this graph directly (i.e., without using the uncompressed de Bruijn graph) based on a suffix tree, where *n* is the total length of the genomes and *g* is the length of the longest genome. Baier et al. (Bioinformatics 32:497–504, 2016) improved their result.

**Results:**

In this paper, we propose a new space-efficient representation of the compressed de Bruijn graph that adds the possibility to search for a pattern (e.g. an allele—a variant form of a gene) within the pan-genome. The ability to search within the pan-genome graph is of utmost importance and is a design goal of pan-genome data structures.

## Background

Nowadays, next generation sequencers produce vast amounts of DNA sequence information and it is often the case that multiple genomes of the same or closely related species are available. An example is the 1000 Genomes Project, which started in 2008. Its goal was to sequence the genomes of at least 1000 humans from all over the world and to produce a catalog of all variations (SNPs, indels, etc.) in the human population. The genomic sequences together with this catalog is called the “pan-genome” of the population. There are several approaches that try to capture variations between many individuals/strains in a population graph; see e.g. [1–4]. These works all require a multi-alignment as input. By contrast, Marcus et al. [5] use a compressed de Bruijn graph of maximal exact matches (MEMs) as a graphical representation of the relationship between genomes; this is basically a compressed version of the colored de Bruijn graph introduced in [6]. They describe an $O(n\log g)$ time algorithm that directly computes the compressed de Bruijn graph on a suffix tree, where *n* is the total length of the genomes and *g* is the length of the longest genome. Marcus et al. write in [5, Section 4]: “Future work remains to improve splitMEM and further unify the family of sequence indices. Although ..., most desired are techniques to reduce the space consumption ...”

In [7] we presented two algorithms that construct the compressed de Bruijn graph using significantly less space (two orders of magnitude) and are faster than splitMEM: $\textsf {A1}$ with run time complexity $O(n\log n)$ and a linear-time algorithm called $\textsf {A2}$. We also mentioned a third algorithm $\textsf {A3}$ that reduces the space requirements further and needs $O(n\log \sigma )$ time, where $\sigma$ is the size of the underlying alphabet. All of the three algorithms $\textsf {A1}$–$\textsf {A3}$ use an FM-index of the genomes and in practice $\textsf {A1}$ is the fastest and $\textsf {A3}$ is the most space-efficient algorithm among them. In [8] we presented $\textsf {A3}$ in detail together with a novel linear-time algorithm based on a compressed suffix tree. In a comparison, it turned out that $\textsf {A3}$ requires less memory and is faster than the algorithm based on the compressed suffix tree. In this article, we present a modification of $\textsf {A1}$ that was inspired by $\textsf {A3}$. We call this new algorithm $\textsf {A4}$ and for the reader’s convenience we decided to explain it in full detail. The *main contribution* of this paper is a new space-efficient representation of the compressed de Bruijn graph that can be calculated with $\textsf {A4}$. This new representation adds the possibility to search for a pattern (e.g. an allele—a variant form of a gene) within the pan-genome. More precisely, one can use the FM-index to search for the pattern and, if the pattern occurs in the pan-genome, one can start the exploration of the compressed de Bruijn graph at the nodes that correspond to the pattern. The ability to search within the pan-genome graph is of utmost importance and is stated as a design goal of the data structure [9, p. 10]. Thus this article constitutes a significant improvement on previous work. We expect that the implicit representation will totally replace the explicit representation. If for some reason an application will require both representations, the implicit representation can easily be transformed into the explicit representation (i.e., we also provide an efficient algorithm for this task). Finally, we show that the memory requirement of $\textsf {A4}$ can be further reduced by using compressed bit vectors.

**Fig. 1:**
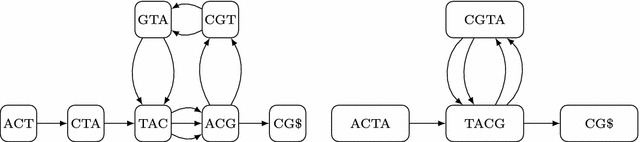
The de Bruijn graph for $$k=3$$ and the string ACTACGTACGTACG$ is shown on the *left*, while its compressed counterpart is shown on the *right*

The de Bruijn graph is also used in sequence assembly where an unknown long DNA sequence is reconstructed from a set of *k*-mers (strings of length *k*). A de Bruijn graph of order *k* stores a *k*-mer in each node. In sequence assembly, the de Bruijn graph has an edge between two nodes if the corresponding *k*-mers overlap exactly $k-1$ characters. In this paper, all *k*-mers originate from several longer strings and two nodes are connected by an edge if the corresponding *k*-mers occur consecutively in one of the strings. The difference can be seen in Fig. 1 for $k=3$: Although TAC and ACT overlap $k-1$ characters, there is no edge between them because they do not occur consecutively in the string. Therefore, algorithms that construct de Bruijn graphs for assembly (see e.g. [10–13] and references therein) can not be used for our problem and vice versa.

Recently, [14] suggested to use a Bloom Filter Trie for pan-genome storage: They use a colored de Bruijn graph, where each stored *k*-mer has a color, but the condition for an edge is the same as in sequence assembly, i.e., the presence of an edge is independent of the color and the origin of the corresponding *k*-mers. Consequently, their algorithm can not be used in our application.

The *contracted* de Bruijn graph introduced by Cazaux et al. [15] is closely related but not identical to the compressed de Bruijn graph. A node in the contracted de Bruijn graph is not necessarily a substring of one of the genomic sequences (see the remark following Definition 3 in [15]). Thus the contracted de Bruijn graph, which can be constructed in linear time from the suffix tree [15], is not useful for our purposes.

## Preliminaries

**Table 1:** Index data structures of the string ACTACGTACGTACG$

*i*	$$\mathsf {SA}$$	$$\mathsf {LCP}$$	$$B_{r}$$	$$B_{l}$$	$$\mathsf {LF}$$	$$\Psi $$	$$\mathsf {BWT}$$	$$S_{\mathsf {SA}[i]}$$
1	15	−1	0	0	10	5	G	$
2	12	0	1	0	13	6	T	ACG$
3	8	3	0	0	14	7	T	ACGTACG$
4	4	7	1	0	15	8	T	ACGTACGTACG$
5	1	2	0	0	1	9	$	ACTACGTACGTACG$
6	13	0	0	0	2	10	A	CG$
7	9	2	0	0	3	11	A	CGTACG$
8	5	6	0	0	4	12	A	CGTACGTACG$
9	2	1	0	1	5	15	A	CTACGTACGTACG$
10	14	0	0	0	6	1	C	G$
11	10	1	0	0	7	13	C	GTACG$
12	6	5	0	1	8	14	C	GTACGTACG$
13	11	0	0	0	11	2	G	TACG$
14	7	4	0	0	12	3	G	TACGTACG$
15	3	8	0	0	9	4	C	TACGTACGTACG$
16		−1						

The suffix array $$\mathsf {SA}$$ of the string ACTACGTACGTACG$ and related notions are defined in section "Preliminaries". The bit vectors $$B_r$$ and $$B_l$$ for $$k=3$$ are explained in section “Computation of right-maximal k-mers and node identifiers”

Let $\Sigma$ be an ordered alphabet of size $\sigma$ whose smallest element is the sentinel character ${\$}$. In the following, *S* is a string of length *n* on $\Sigma$ having the sentinel character at the end (and nowhere else). In pan-genome analysis, *S* is the concatenation of multiple genomic sequences, where the different sequences are separated by special symbols (in practice, we use one separator symbol and treat the different occurrences of it as if they were different characters; see “Computation of right-maximal k-mers and node identifiers” section). For $1 \le i \le n$, *S*[*i*] denotes the *character at positioni* in *S*. For $i \le j$, *S*[*i*..*j*] denotes the *substring* of *S* starting with the character at position *i* and ending with the character at position *j*. Furthermore, $S_i$ denotes the *i*-th suffix *S*[*i*..*n*] of *S*. The *suffix array*$\mathsf {SA}$ of the string *S* is an array of integers in the range 1 to *n* specifying the lexicographic ordering of the *n* suffixes of *S*, that is, it satisfies $S_{\mathsf {SA}[1]}< S_{\mathsf {SA}[2]}< \cdots < S_{\mathsf {SA}[n]};$ see Table 1 for an example. A suffix array can be constructed in linear time; see e.g. the overview article [16]. For every substring $\omega$ of *S*, the $\omega$-interval is the suffix array interval [*i*..*j*] so that $\omega$ is a prefix of $S_{\mathsf {SA}[k]}$ if and only if $i\le k \le j$.

The Burrows–Wheeler transform [17] converts *S* into the string $\mathsf {BWT}[1..n]$ defined by $\mathsf {BWT}[i]=S[\mathsf {SA}[i] -1]$ for all *i* with $\mathsf {SA}[i] \ne 1$ and $\mathsf {BWT}[i] = {\$}$ otherwise; see Table 1. Several semi-external and external memory algorithms are known that construct the $\mathsf {BWT}$ directly (i.e., without constructing the suffix array); see e.g. [18–21].

The *wavelet tree* [22] of the $$\mathsf {BWT}$$ supports one backward search step in $$O(\log \sigma )$$ time [23]: Given the $$\omega $$-interval [*lb*..*rb*] and a character *c* from the alphabet $$\Sigma $$, *backwardSearch*(*c*, [*lb*..*rb*]) returns the $$c\omega $$-interval [*i*..*j*] (i.e., $$i\le j$$ if $$c\omega $$ is a substring of *S*; otherwise $$i > j$$). This crucially depends on the fact that a bit vector *B* can be preprocessed in linear time so that an arbitrary $$rank_1(B,i)$$ query (asks for the number of ones in *B* up to and including position *i*) can be answered in constant time [24]. Backward search can be generalized on the wavelet tree as follows: Given an $$\omega $$-interval [*lb*..*rb*], a slight modification of the procedure *getIntervals*([*lb*..*rb*]) described in [25] returns the list $$[(c,[i..j]) \mid c\omega \text{ is } \text{ a } \text{ substring } \text{ of } S \text{ and } [i..j] \text{ is } \text{ the } $$$$c\omega \text{-interval}]$$, where the first component of an element (*c*, [*i*..*j*]) must be a character. The worst-case time complexity of the procedure *getIntervals* is $$O(z + z \log (\sigma /z))$$, where *z* is the number of elements in the output list; see [26, Lemma 3].

The $\mathsf {LF}$-mapping (last-to-first-mapping) is defined as follows: If $\mathsf {SA}[i] = q$, then $\mathsf {LF}(i)$ is the index *j* so that $\mathsf {SA}[j] = q-1$ (if $\mathsf {SA}[i] = 1$, then $\mathsf {LF}(i)=1$). In other words, if the *i*-th entry in the suffix array is the suffix $S_q$, then $\mathsf {LF}(i)$ “points” to the entry at which the suffix $S_{q-1}$ can be found; see Table 1. The function $\Psi$ is the inverse of the $\mathsf {LF}$-mapping. Using the wavelet tree of the $\mathsf {BWT}$, a value $\mathsf {LF}(i)$ or $\Psi (i)$ can be calculated in $O(\log \sigma )$ time. For later purposes, we recall how the $\mathsf {LF}$-mapping can be computed from the $\mathsf {BWT}$. First, the *C*-array is calculated, where for each $c \in \Sigma$, *C*[*c*] is the overall number of occurrences of characters in $\mathsf {BWT}$ that are strictly smaller than *c*. Second, if in a left-to-right scan of the $\mathsf {BWT}$, where the loop-variable *i* varies from 1 to *n*, *C*[*c*] is incremented by one for $c = \mathsf {BWT}[i]$, then $\mathsf {LF}[i] = C[c]$.

The suffix array $\mathsf {SA}$ is often enhanced with the so-called $\mathsf {LCP}$-array containing the lengths of longest common prefixes between consecutive suffixes in $\mathsf {SA}$; see Table 1. Formally, the $\mathsf {LCP}$-array is an array so that $\mathsf {LCP}[1] = -1 = \mathsf {LCP}[n+1]$ and $\mathsf {LCP}[i] = \left|\mathsf {lcp}(S_{\mathsf {SA}[i-1]},S_{\mathsf {SA}[i]})\right|$ for $2\le i \le n$, where $\mathsf {lcp}(u,v)$ denotes the longest common prefix between two strings *u* and *v*. The $\mathsf {LCP}$-array can be computed in linear time from the suffix array and its inverse, but it is also possible to construct it directly from the wavelet tree of the $\mathsf {BWT}$ in $O(n \log \sigma )$ time with the help of the procedure *getIntervals* [25].

A substring $\omega$ of *S* is a *repeat* if it occurs at least twice in *S*. Let $\omega$ be a repeat of length $\ell$ and let [*i*..*j*] be the $\omega$-interval. The repeat $\omega$ is *left-maximal* if $|\{\mathsf {BWT}[x] \mid i \le x \le j\}| \ge 2$, i.e., the set $\{S[\mathsf {SA}[x]-1] \mid i \le x \le j\}$ of all characters that precede at least one of the suffixes $S_{\mathsf {SA}[i]},\dots ,S_{\mathsf {SA}[j]}$ is not singleton (where $S[0] := \$$). Analogously, the repeat $\omega$ is *right-maximal* if $|\{S[\mathsf {SA}[x]+\ell ] \mid i \le x \le j\}| \ge 2$. A left- and right-maximal repeat is called *maximal* repeat. (Note that [5] use the term “maximal exact match” instead of the more common term “maximal repeat”. We will not use the term “maximal exact match” here). A detailed explanation of the techniques used here can be found in [27].

## Compressed de Bruijn graph

Given a string *S* of length *n* and a natural number *k*, the de Bruijn graph of *S* contains a node for each distinct length *k* substring of *S*, called a *k*-mer. Two nodes *u* and *v* are connected by a directed edge (*u*, *v*) if *u* and *v* occur consecutively in *S*, i.e., $u = S[i..i+k-1]$ and $v = S[i+1..i+k]$. Fig. 1 shows an example. Clearly, the graph contains at most *n* nodes and *n* edges. By construction, adjacent nodes will overlap by $k-1$ characters, and the graph can include *multiple* edges connecting the same pair of nodes or self-loops representing overlapping repeats. For every node, except for the start node (containing the first *k* characters of *S*) and the stop node (containing the last *k* characters of *S*), the in-degree coincides with the out-degree. A de Bruijn graph can be “compressed” by merging non-branching chains of nodes into a single node with a longer string. More precisely, if node *u* is the only predecessor of node *v* and *v* is the only successor of *u* (but there may be multiple edges (*u*, *v*)), then *u* and *v* can be merged into a single node that has the predecessors of *u* and the successors of *v*. After maximally compressing the graph, every node (apart from possibly the start node) has at least two different predecessors or its single predecessor has at least two different successors and every node (apart from the stop node) has at least two different successors or its single successor has at least two different predecessors; see Fig. 1. Of course, the compressed de Bruijn graph can be built from its uncompressed counterpart (a much larger graph), but this is disadvantageous because of the huge space consumption. That is why we will build it directly.

**Fig. 2:**
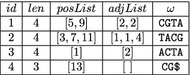
Explicit representation of the compressed de Bruijn graph from Fig. 1

Figure 2 shows how splitMEM represents the compressed de Bruijn graph *G* for $$k=3$$ and the string $$S=$$ ACTACGTACGTACG$. Each node corresponds to a substring $$\omega $$ of *S* and consists of the components (*id*, *len*, *posList*, *adjList*), where *id* is a natural number that uniquely identifies the node, *len* is the length $$|\omega |$$ of $$\omega $$, *posList* is the list of positions at which $$\omega $$ occurs in *S* (sorted in ascending order), and *adjList* is the list of the successors of the node (sorted in such a way that the walk through *G* that gives *S* is induced by the adjacency lists: if node *G*[*id*] is visited for the *i*-th time, then its successor is the node that can be found at position *i* in the adjacency list of *G*[*id*]).

The nodes in the compressed de Bruijn graph of a pan-genome can be categorized as follows:

- A uniqueNode represents a unique substring in the pan-genome and has a single start position (i.e., *posList* contains just one element)
- A repeatNode represents a substring that occurs at least twice in the pan-genome, either as a repeat in a single genome or as a segment shared by multiple genomes.

In pan-genome analysis, *S* is the concatenation of multiple genomic sequences, where the different sequences are separated by a special symbol $\#$. (In theory, one could use pairwise different symbols to separate the sequences, but in practice this would blow up the alphabet.) This has the effect that $\#$ may be part of a repeat. In contrast to splitMEM, our algorithm treats the different occurrences of $\#$ as if they were different characters. Consequently, $\#$ will not be a part of a repeat. In our approach, each occurrence of $\#$ will be the end of a stop node (i.e., there is a stop node for each sequence).

According to [5], the compressed de Bruijn graph is most suitable for pan-genome analysis: “This way the complete pan-genome will be represented in a compact graphical representation such that the shared/strain-specific status of any substring is immediately identifiable, along with the context of the flanking sequences. This strategy also enables powerful topological analysis of the pan-genome not possible from a linear representation.” It has one defect though: it is not possible to search efficiently for certain nodes and then to explore the graph in the vicinity of these nodes. A user might, for example, want to search for a certain allele in the pan-genome and—if it is present—to examine the neighborhood of that allele in the graph. Here, we propose a new space-efficient representation of the compressed de Bruijn graph that adds exactly this functionality.

We store the graph in an array *G* of length *N*, where *N* is the number of nodes in the compressed de Bruijn graph. Moreover, we assign to each node a unique identifier $id \in \{1,\dots ,N\}$. A node *G*[*id*] now has the form $(len,lb,size,{ suffix}\_lb)$, where

- The variable *len* is the length of the string $\omega = S[\mathsf {SA}[lb]..\mathsf {SA}[lb]+len-1]$ that corresponds to the node with identifier *id*
- $[lb..lb+size-1]$ is the $\omega$-interval, *lb* is its left boundary, and *size* is its size
- $[{ suffix}\_lb..{ suffix}\_lb+size-1]$ is the interval of the *k* length suffix of $\omega$

There is one exception though: the sentinel $ and each occurrence of the separator $$\#$$ will be the end of a stop node. Clearly, the suffix $ of *S* appears at index 1 in the suffix array because $ is the smallest character in the alphabet. The suffix array interval of $ is [1..1], so we set $${ suffix}\_lb= 1$$. Analogously, a suffix of *S* that starts with $$\#$$ appears at an index $$j \in \{2,\dots ,d\}$$ in the suffix array (where $$d$$ is the number of sequences in *S*) because $$\#$$ is the second smallest character in the alphabet, so we set $${ suffix}\_lb= j$$.

**Fig. 3:**
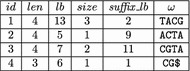
Implicit representation of the compressed de Bruijn graph from Fig. 1

Figure 3 shows an example. Henceforth this representation will be called implicit representation, while the representation from Fig. 2 will be called explicit representation. It is clear that in the implicit representation the list of all positions at which $\omega$ occurs in *S* can be computed as follows: $[\mathsf {SA}[i] \mid lb \le i \le lb+size-1]$. It will be explained later, how the graph can be traversed and how a pattern can be searched for. We shall see that this can be done efficiently by means of the fourth component ${ suffix}\_lb$.

## Construction algorithm

We will build the implicit representation of the compressed de Bruijn graph directly from an FM-index (the wavelet tree of the $\mathsf {BWT}$) of *S*, using Lemma 1 (the simple proof is omitted).

### **Lemma 1**

*Letvbe a node in the compressed de Bruijn graph and let *$\omega$*be the string corresponding to v*.* If v is not the start node, then it has at least two different predecessors if and only if the lengthkprefix of*$\omega$*is a left-maximal repeat. It has at least two different successors if and only if the lengthksuffix of *$\omega$*is a right-maximal repeat.*

**Fig. 4:**
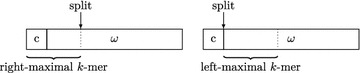
The string $\omega$ must be split if the length *k* prefix of $c\omega$ is a *right-maximal* repeat or the length *k* prefix of $\omega$ is a *left-maximal* repeat

The general idea behind our algorithm is as follows. Compute the suffix array intervals of all right-maximal *k*-mers. For each such *k*-mer *v*, compute all *cv*-intervals, where $c\in \Sigma$. Then, for each $u=cv$, compute all *bu*-intervals, where $b\in \Sigma$, etc. In other words, we start with all right-maximal *k*-mers and extend them as long as possible (and in all possible ways with the procedure *getIntervals*), character by character, to the left. According to Lemma 1, the left-extension of a string $\omega$ must stop if (i) the length *k* prefix of $\omega$ is a left-maximal repeat (this is the case if the procedure *getIntervals* applied to the $\omega$-interval returns a non-singleton list). It must also stop if (ii) the length *k* prefix *v* of $c\omega$ is a right-maximal repeat for some $c\in \Sigma$; see Fig. 4. This is because by Lemma 1 there is a node *uv*, $u\in \Sigma ^*$, in the compressed de Bruijn graph with at least two different successors (the length *k* suffix *v* of *uv* is a right-maximal repeat). Consequently, there must be a directed edge $(uv,\omega )$ in the compressed de Bruijn graph. In the following, we will explain the different phases of the algorithm in detail.

### Computation of right-maximal *k*-mers and node identifiers

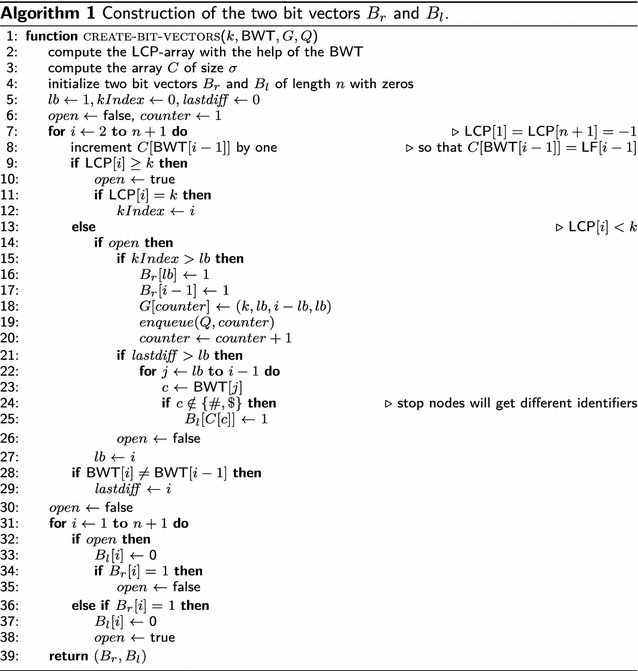



As explained above, there is a directed edge $(v,\omega )$ in the compressed de Bruijn graph if the *k*-length suffix of *v* is a right-maximal repeat or the *k*-length prefix of $\omega$ is a left-maximal repeat. Since we proceed from right to left, we must be able to compute an identifier *id* of the node corresponding to *v*. In case that the *k*-length suffix of *v* is a right-maximal repeat, we will use the bit vector $B_{r}$ to compute the identifier *id*. $B_{r}$ has the property that $B_{r}[i]=1$ if and only if the *k*-mer $u=S[\mathsf {SA}[i]..\mathsf {SA}[i]+k-1]$ is a right-maximal repeat and $S_{\mathsf {SA}[i]}$ is either the lexicographically smallest or the lexicographically largest suffix of *S* that has *u* as a prefix (in other words, the left- and right boundary of the *u*-interval in the suffix array is marked by a 1 in $B_{r}$). In case that the *k*-length suffix of *v* is not a right-maximal repeat but the *k*-length prefix of $\omega$ is a left-maximal repeat, we will use the bit vector $B_{l}$ to compute the identifier *id*. $B_{l}$ has the property that $B_{l}[i]=1$ if and only if (i) the *k*-mer $u=S[\mathsf {SA}[i]..\mathsf {SA}[i]+k-1]$ is *not* a right-maximal repeat, (ii) $S_{\mathsf {SA}[i]}$ is the lexicographically largest suffix of *S* that has *u* as a prefix (in other words, *i* is the right boundary of the *u*-interval in the suffix array), *and* the *k*-mer $S[\mathsf {SA}[i]+1..\mathsf {SA}[i]+k]$ is a left-maximal repeat.

To obtain the bit vector $B_{r}$, we must compute all right-maximal *k*-mers and their suffix array intervals. Let *u* be a right-maximal *k*-mer and consider the *u*-interval [*lb*..*rb*] in the suffix array. Note that (1) $\mathsf {LCP}[lb] < k$ and (2) $\mathsf {LCP}[rb+1] < k$. Since *u* is right-maximal, *u* is the longest common prefix of all suffixes in the interval [*lb*..*rb*]. This implies (3) $\mathsf {LCP}[j] \ge k$ for all *j* with $lb+1\le j \le rb$ and (4) $\mathsf {LCP}[j] = k$ for at least one *j* with $lb+1\le j \le rb$ (in the terminology of [28], [*lb*..*rb*] is an lcp-interval of lcp-value *k*). It follows as a consequence that the bit vector $B_{r}$ can be calculated with the help of the $\mathsf {LCP}$-array. Using the algorithm of [25], Algorithm 1 constructs the $\mathsf {LCP}$-array directly from the $\mathsf {BWT}$ in $O(n \log \sigma )$ time, where $\sigma$ is the size of the alphabet. It is not difficult to verify that lines 9–17 of Algorithm 1 compute all suffix array intervals of right-maximal *k*-mers. Furthermore, on lines 16 and 17 the boundaries *lb* and $rb=i-1$ of the *k*-mer intervals are marked by setting the entries of $B_{r}$ at these positions to 1. On line 18, the node $(lb,k,i-lb,lb)$ having the current value of the variable *counter* as identifier is added to the graph *G*. In contrast to the last two components, the first two components of a node may change later (they will change when a left-extension is possible). On line 19, the node identifier is added to the queue *Q* and then *counter* is incremented by one.

We would like to stress that all right-maximal *k*-mers can be determined without the entire $\mathsf {LCP}$-array. In order to verify whether or not an interval satisfies properties (1)–(4), it is sufficient to compute all entries ≤ *k* in the $\mathsf {LCP}$-array (the others have a value > *k*). Since the algorithm of [25] calculates entries in the $\mathsf {LCP}$-array in ascending order, it is ideal for our purposes. We initialize an array *L* with values 2 and set $L[1]=0$ and $L[n+1]=0$. Two bits are enough to encode the case “$< k$” by 0, the case “$= k$” by 1, and the case “$> k$” by 2 (so initially all entries in the $\mathsf {LCP}$-array are marked as being $>k$, except for *L*[1] and $L[n+1]$, which are marked as being $<k$). Then, for $\ell$ from 0 to $k-1$, the algorithm of [25] calculates all indices *p* with entries $\mathsf {LCP}[p] = \ell$ and sets $L[p] = 0$. Furthermore, it continues to calculate all indices *q* with entries $\mathsf {LCP}[q] = k$ and sets $L[q] = 1$. Now the array *L* contains all the information that is needed to compute right-maximal *k*-mers.

As already mentioned, in pan-genome analysis $S=S^1\#S^2\#\dots S^{d-1}\#S^d\$$ is the concatenation of multiple genomic sequences $S^1,\dots ,S^d$, separated by a special symbol $\#$. Our algorithm treats the different occurrences of $\#$ as if they were different characters. Assuming that $\#$ is the second smallest character, this can be achieved as follows. As explained above, all right-maximal *k*-mers can be determined without the entire $\mathsf {LCP}$-array if the algorithm in [25] is used. If there are $d-1$ occurrences of $\#$ in total and this algorithm starts with $d-1$ singleton intervals [*s*..*s*], $2\le s \le d$, instead of the $\#$-interval $[2..d]$, then the different occurrences of $\#$ are treated as if they were different characters.

Bit vector $$B_{l}$$ is computed on lines 7–29 of Algorithm 1 as follows: If the suffix array interval [*lb*..*rb*] of a repeat $$\omega $$ of length $$\ge k$$ is detected, then it must be checked whether or not $$\omega $$ is left-maximal (note that $$rb= i-1$$). Recall that $$\omega $$ is a left-maximal repeat if and only if $$|\{\mathsf {BWT}[lb],\mathsf {BWT}[lb+1],\dots , \mathsf {BWT}[rb]\}| \ge 2$$. Algorithm 1 checks this condition by keeping track of the largest index $$ lastdiff $$ at which the characters $$\mathsf {BWT}[ lastdiff -1]$$ and $$\mathsf {BWT}[ lastdiff ]$$ differ; see lines 28 and 29. Since $$ lastdiff \le rb= i-1$$, the characters $$\mathsf {BWT}[lb],\mathsf {BWT}[lb+1],\dots , \mathsf {BWT}[rb]$$ are not all the same if and only if $$ lastdiff > lb$$. If this condition on line 21 evaluates to true, then for each $$c\notin \{\#,\$\}$$ in $$\mathsf {BWT}[lb..rb]$$ the algorithm sets $$B_{l}[\mathsf {LF}[q]]$$ to 1 in lines 23–25, where *q* is the index of the last occurrence of $$c \in \mathsf {BWT}[lb..rb]$$ and $$\mathsf {LF}$$ is the last-to-first mapping. How this is done by means of the *C*-array will be explained below. So a one in $$B_{l}$$ marks a *k*-mer that precedes a left-maximal *k*-mer. Since we are only interested in those *k*-mers that are not right-maximal (right-maximal *k*-mers are already covered by bit vector $$B_{r}$$), lines 30–38 of Algorithm 1 reset those one-bits in $$B_{l}$$ to zero that mark a right-maximal *k*-mer.

It remains for us to explain the computation of the $B_{l}$ vector with the *C*-array. After the computation of the *C*-array on line 3 of Algorithm 1, for each $c \in \Sigma$, *C*[*c*] is the overall number of occurrences of characters in *S* that are strictly smaller than *c*. Moreover, after line 8 of Algorithm 1 was executed, we have $C[\mathsf {BWT}[i-1]]=\mathsf {LF}[i-1]$ (to see this, recall from "Preliminaries" section how the $\mathsf {LF}$-mapping can be computed from the $\mathsf {BWT}$). Thus, when the for-loop on lines 7–29 of Algorithm 1 is executed for a certain value of *i*, we have $C[c]=\mathsf {LF}[q]$ for each character *c* in $\mathsf {BWT}[1..i-1]$, where *q* is the index of the last occurrence of *c* in $\mathsf {BWT}[1..i-1]$. Algorithm 1 uses this fact on line 25: $C[c]=\mathsf {LF}[q]$, where *q* is the index of the last occurrence of *c* in $\mathsf {BWT}[lb..i-1]$.

Apart from the direct construction of the $\mathsf {LCP}$-array from the $\mathsf {BWT}$, which takes $O(n \log \sigma )$ time, Algorithm 1 has a linear run-time. The overall run-time is therefore $O(n \log \sigma )$.

### Construction of the space-efficient representation

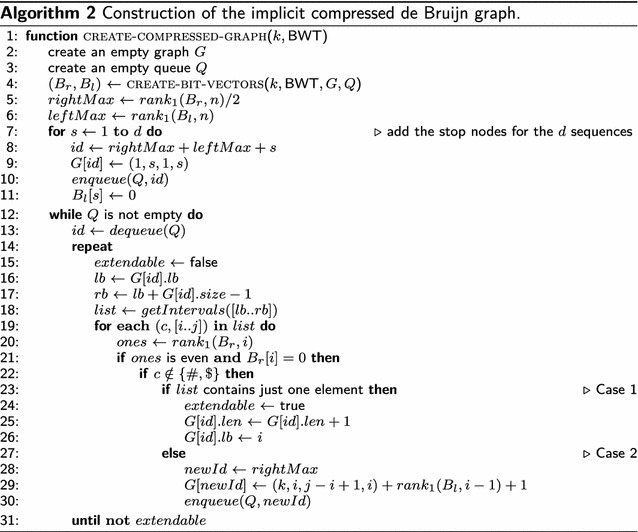



Algorithm 2 constructs the implicit representation of the compressed de Bruijn graph. It calls Algorithm 1, which computes—besides the two bit vectors $B_{r}$ and $B_{l}$—the suffix array interval $[lb..lb+size-1]$ of each right-maximal *k*-mer $\omega$, stores the quadruple (*k*, *lb*, *size*, *lb*) at *G*[*id*], where $id = (rank_1(B_{r},lb)+1)/2$ (this is because Algorithm 1 computes right-maximal *k*-mer intervals in lexicographical order), and adds *id* to the (initially empty) queue *Q*. The attributes *G*[*id*].*size* and $G[id].{ suffix}\_lb$ will never change, but the attributes *G*[*id*].*len* and *G*[*id*].*lb* will change when a left-extension is possible. In the for-loop on lines 7–11, the stop nodes are added to *G* and their identifiers are added to *Q*. In the while-loop on lines 12–31, as long as the queue *Q* is not empty, the algorithm removes an identifier *id* from *Q* and in a repeat-loop computes $list = getIntervals([lb..rb])$, where $lb=G[id].lb$ and $rb= lb + G[id].size - 1$. During the repeat-loop, the interval [*lb*..*rb*] is the suffix array interval of some string $\omega$ of length *G*[*id*].*len*. In the body of the repeat-loop, a flag *extendable* is set to false. The procedure call *getIntervals*([*lb*..*rb*]) then returns the list *list* of all $c\omega$-intervals. At this point, the algorithm tests whether or not the length *k* prefix of $c\omega$ is a right-maximal repeat. It is not difficult to see that the length *k* prefix of $c\omega$ is a right-maximal repeat if and only if the $c\omega$-interval [*i*..*j*] is a subinterval of a right-maximal *k*-mer interval. Here, the bit vector $B_{r}$ comes into play. At the beginning of Algorithm 2, all suffix array intervals of right-maximal *k*-mers have been computed and their boundaries have been marked in $B_{r}$. It is crucial to note that these intervals are disjoint. Lemma 2 shows how the bit vector $B_{r}$ can be used to test for non-right-maximality.

#### **Lemma 2**

*The *$c\omega$*-interval *[*i*..*j*]* is not a subinterval of a right-maximal k-mer interval if and only if *$rank_1(B_{r},i)$*, the number of ones in *$B_{r}$*up to (and including) positioni, is even and *$B_{r}[i] = 0$.

#### *Proof*

“Only-if:” Suppose [*i*..*j*] is not a subinterval of a right-maximal *k*-mer interval. Since [*i*..*j*] cannot overlap with a right-maximal *k*-mer interval, it follows that $rank_1(B_{r},i)$ must be even and $B_{r}[i..j]$ contains only zeros.

“if:” Suppose [*i*..*j*] is a subinterval of a right-maximal *k*-mer interval [*p*..*q*]. If $i\ne j$, then $rank_1(B_{r},i)$ must be odd. If $i = j$, then $rank_1(B_{r},i)$ may be even. But in this case *i* must be the right boundary of the interval [*p*..*q*], so $B_{r}[i] = B_{r}[q] =1$.$\hfill\square$

Now, the algorithm proceeds by case analysis. If the length *k* prefix of $c\omega$ is a right-maximal repeat, there must be a node *v* that ends with the length *k* prefix of $c\omega$ (note that $c\omega [1..k]$ and $\omega$ have a suffix-prefix-overlap of $k-1$ characters), and this node *v* will be detected by a computation that starts with the *k*-mer $c\omega [1..k]$. Consequently, the computation stops here. If the length *k* prefix of $c\omega$ is not a right-maximal repeat, one of the following two cases occurs:

1. If *list* contains just one element (*c*, [*i*..*j*]), then $\omega$ is not left-maximal. In this case, the algorithms sets *extendable* to true, *G*[*id*].*lb* to *i*, and increments *G*[*id*].*len* by one. Now *G*[*id*] represents the $c\omega$-interval [*i*..*j*] and the repeat-loop continues with this interval. Note that $G[id].size = j-i+1$ because $\omega$ is not left-maximal.
2. Otherwise, $\omega$ is left-maximal. In this case, a split occurs (so the attributes of *G*[*id*] will not change any more) and Algorithm 2 must continue with the *k*-mer prefix $x=c\omega [1..k]$ of $c\omega$. For the correctness of the algorithm, it is important to note that the interval [*i*..*j*] is the *x*-interval; see Lemma 3. We use the bit vector $B_{l}$ to assign the unique identifier $newId=rightMax + rank_1(B_{l},i-1)+1$ to the next node, which corresponds to (or ends with) *x* (recall that *rightMax* is the number of all right-maximal *k*-mers and that *x* is not a right-maximal *k*-mer). So a quadruple $(k,i,j-i+1,i)$ is inserted at *G*[*newId*] and *newId* is added to *Q*.

#### **Lemma 3**

*Consider the *$c\omega$*-interval* [*i*..*j*]* in Case 2 of Algorithm 2 (beginning at line 27). The interval *[*i*..*j*] *coincides with the *$c\omega [1..k]$*-interval* [*p*..*q*].

#### *Proof*

Clearly, [*i*..*j*] is a subinterval of [*p*..*q*] because $c\omega [1..k]$ is a prefix of $c\omega$. For a proof by contradiction, suppose that $[i..j] \ne [p..q]$. Let *cu* be the longest common prefix of all suffixes in the interval [*p*..*q*]. Note that the length $\ell$ of *cu* is at least *k*. Since $[i..j] \ne [p..q]$, it follows that there must be a suffix in the interval [*p*..*q*] that has a prefix *cub* so that *cu* is a proper prefix of $c\omega$ and $b\ne c\omega [\ell +1]$. Consequently, *cu* is a right-maximal repeat. Clearly, this implies that *u* is a right-maximal repeat as well. We consider two cases:

1. $\ell =k$: In this case, Algorithm 2 stops (the length *k* prefix *cu* of $c\omega$ is a right-maximal repeat), so it cannot execute Case 2; a contradiction.
2. $\ell > k$: Note that *u* has length $\ell -1 \ge k$. Since *u* is a right-maximal repeat, it is impossible that the procedure *getIntervals* is applied to the $\omega$-interval [*lb*..*rb*]. This contradiction proves the Lemma.$\hfill\square$

As an example, we apply Algorithm 2 to $$k=3$$ and the $$\mathsf {LCP}$$-array and the $$\mathsf {BWT}$$ of the string ACTACGTACGTACG$; see Table 1. There is only one right maximal *k*-mer, ACG, so a node $$(len,lb,size,{ suffix}\_lb) = (3,2,3,2)$$ is inserted at *G*[1] and the identifier 1 is added to the queue *Q* in Algorithm 1. On line 9 of Algorithm 2 the stop node is added to *G*. It has the identifier $$rightMax+leftMax+1 = 1+2+1=4$$, so *G*[4] is set to (1, 1, 1, 1) and 4 is added to *Q*. In the while-loop, the identifier 1 of node (3, 2, 3, 2) is dequeued and the procedure call *getIntervals*([2..4]) returns a list that contains just one interval, the TACG-interval [13..15]. Since $$rank_1(B_{r},13) = 2$$ is even and $$B_{r}[13]=0$$, Case 1 applies. So *extendable* is set to true and *G*[1] is modified to (4, 13, 3, 2). In the next iteration of the repeat-loop, *getIntervals*([13..15]) returns the list $$[(\texttt {C},[9..9]), (\texttt {G},[11..12])]$$, where [9..9] is the CTACG-interval and [11..12] is the GTACG-interval. It is readily verified that Case 2 applies in both cases. For the CTACG-interval [9..9] we obtain the identifier $$rightMax+rank_1(B_{l},9-1)+1 = 1+0+1=2$$, so *G*[2] is set to (3, 9, 1, 9). Analogously, the GTACG-interval [11..12] gets the identifier $$rightMax+rank_1(B_{l},11-1)+1 = 1+1+1=3$$ and *G*[3] is set to (3, 11, 2, 11). Furthermore, the identifiers 2 and 3 are added to the queue *Q*. Next, the identifier 4 of the stop node (1, 1, 1, 1) is dequeued and the procedure call *getIntervals*([1..1]) returns a list that contains just one interval, the G$-interval [10..10]. Case 1 applies, so *G*[4] is modified to (2, 10, 1, 1). In the second iteration of the repeat-loop, *getIntervals*([10..10]) returns the CG$-interval [6..6]. Again Case 1 applies and *G*[4] is modified to (3, 6, 1, 1). In the third iteration of the repeat-loop, *getIntervals*([6..6]) returns the ACG$-interval [2..2]. This time, $$rank_1(B_{r},2)=1$$ is odd and therefore the repeat-loop terminates. The computation continues until the queue *Q* is empty; the final compressed de Bruijn graph is shown in Fig. 3.

We claim that Algorithm 2 has a worst-case time complexity of $O(n \log \sigma )$ and use an amortized analysis to prove this. Since the compressed de Bruijn graph has at most *n* nodes, it is an immediate consequence that at most *n* identifiers enter and leave the queue *Q* (this covers Case 2). Case 1 can occur at most *n* times because there are at most *n* left-extensions; so at most *n* intervals generated by the procedure *getIntervals* belong to this category. Each left-extension eventually ends; so at most *n* intervals generated by the procedure *getIntervals* belong to this category because there are at most *n* left-extensions. In summary, at most 2*n* intervals are generated by the procedure *getIntervals*. Since this procedure takes $O(\log \sigma )$ time for each generated interval, the claim follows.

### Construction of the explicit compressed de Bruijn graph


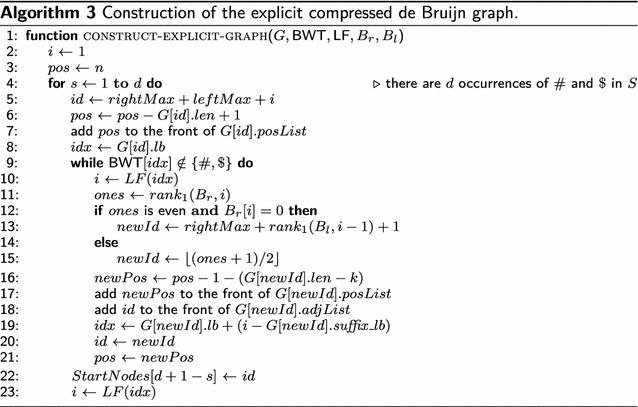
As already mentioned in "Background" section, we expect that the implicit representation will totally replace the explicit representation. In the unexpected case that a future application will require both representations, it might be good to know that the implicit representation can easily be turned into the explicit representation. For this reason, we next describe how this can be done.

 If the pan-genome consists of $d$ sequences, then $S=S^1\#S^2\#\dots S^{d-1}\#S^d\$$ and there are *d* stop nodes. Since the implicit representation allows for an efficient backward traversal, there is no need for start nodes. By contrast, the explicit graph must provide them. That is why Algorithm 3 stores them in an array *StartNodes* of size *d*.

Algorithm 3 starts with the stop node of the last sequence $$S^d$$, which has identifier $$id=rightMax+leftMax+1$$. Let $$\omega $$ be the string corresponding to node *id*. Since $$\omega $$ ends with $ and $ appears at position *n* in *S*, the start position of $$\omega $$ in *S* is $$pos=n-G[id].len +1$$. Consequently, *pos* is added to the front of *G*[*id*].*posList* on line 7 of Algorithm 3. Next, we have to find the predecessor of node *id*. It is not difficult to see that $$idx=G[id].lb$$ is the index in the suffix array at which the suffix $$S_{pos}$$ can be found (note that $$S_{pos}$$ has $$\omega $$ as a prefix). Clearly, $$i=LF(idx)$$ is the index of the suffix $$S_{pos-1}$$ in the suffix array. Note that $$S_{pos-1}$$ has $$c\omega $$ as a prefix, where $$c=\mathsf {BWT}[idx]$$. If *c* is not a separator symbol (i.e., $$c \notin \{\#,{\$}\}$$), then the predecessor of node *id* is the node *newId* whose corresponding string *u* ends with the *k*-mer prefix $$x = c\omega [1..k]$$ of $$c\omega $$. If *x* is a right-maximal *k*-mer, then *newId* is $$(rank_1(B_{r},i)+1)/2$$, otherwise it is $$rightMax + rank_1(B_{l},i-1)+1$$. Note that *u* ends at position $$pos-1+(k-1)$$ in *S* because *u* and $$\omega $$ overlap $$k-1$$ characters. It follows as a consequence that *u* starts at position $$newPos = pos-1+k-1-G[newId].len +1 = pos-1-(G[newId].len - k)$$. So the position *newPos* is added to the front of the position list of *G*[*newId*]. Because node *G*[*id*] is the successor of node *G*[*newId*], the identifier *id* is added to the front of the adjacency list of *G*[*newId*]. To find the predecessor of node *newId* in the same fashion, we must find the index *idx* at which the suffix $$S_{newPos}$$ can be found in the suffix array. According to Lemma 4, this is $$G[newId].lb+(i-G[newId].{ suffix}\_lb)$$. The while-loop repeats the search for a predecessor node until a separator symbol is found. In this case, a start node has been reached and its identifier is stored in an array *StartNodes* of size *d*. Since there are *d* separator symbols, the whole process is executed *d* times.

#### **Lemma 4**

*Let *$G[id] = (len,lb,size,{ suffix}\_lb)$*be a node in the implicit representation of the compressed de Bruijn graph. If G*[*id*] *is not a stop node and suffix *$S_p$*appears at index i in the interval*$[b..e]=[{ suffix}\_lb..{ suffix}\_lb+size-1]$*(i.e., *$\mathsf {SA}[i]=p$*), then the suffix*$S_{p+(len-k)}$*appears at index *$lb+(i-{ suffix}\_lb)$*in the interval*$[lb..lb+size-1]$.

#### *Proof*

Let *u* be the string corresponding to *G*[*id*] and let *x* be the *k*-mer suffix of *u*. By construction, $[lb..lb+size-1]$ is the *u*-interval and [*b*..*e*] is the *x*-interval in the suffix array. If $u=x$, then $len=k$, $lb = { suffix}\_lb$, and there is nothing to show. So suppose $u\ne x$ and let *c* be the character that precedes *x* in *u* (recall that *x* is not left-maximal). Since $S_{\mathsf {SA}[b]}< S_{\mathsf {SA}[b+1]}< \dots < S_{\mathsf {SA}[e]}$, it follows that $cS_{\mathsf {SA}[b]}< cS_{\mathsf {SA}[b+1]}< \dots < cS_{\mathsf {SA}[e]}$. In other words, the *cx*-interval contains the suffixes $S_{\mathsf {SA}[b]-1}< S_{\mathsf {SA}[b+1]-1}< \dots < S_{\mathsf {SA}[e]-1}$. Consequently, if *i* is the *q*-th element of [*b*..*e*] and $\mathsf {SA}[i]=p$, then $\mathsf {LF}(i)$ is the *q*-th element of the *cx*-interval and $\mathsf {SA}[\mathsf {LF}(i)]=p-1$ (this implies in particular that $[\mathsf {LF}(b)..\mathsf {LF}(e)]$ is the *cx*-interval). Iterating this argument $len-k$ times yields the Lemma. $\square$

Algorithm 3 has a worst-case time complexity of $O(N\log \sigma )$, where *N* is the number of edges in the compressed de Bruijn graph. This is because in each execution of the while-loop an edge is added to the graph and a value $\mathsf {LF}(idx)$ is computed in $O(\log \sigma )$ time (all other operations take only constant time). Since the uncompressed de Bruijn graph has at most *n* edges, so does the compressed graph. Hence $N \le n$. In fact, *N* is much smaller than *n* in virtually all cases. It follows from the preceding section that *N* can be characterized in terms of left- and right-maximal *k*-mer repeats. We have seen that the number of nodes in the compressed de Bruijn graph equals $|V_1|+|V_2|+d=rightMax+leftMax+d$, where $V_1 = \{\omega \mid \omega \text{ is } \text{ a } \text{ right-maximal } k\text{-mer } \text{ repeat } \text{ in } S\}$ and $V_2 = \{\omega \mid \exists i \in \{1,\dots ,n-k\} : \omega = S[i..i+k-1] \notin V_1$ and $S[i+1..i+k] \text{ is } \text{ a } \text{ left-maximal } k\text{-mer } \text{ repeat } \text{ in } S\}$; the stop nodes are taken into account by adding *d*. The number *N* of edges in the compressed de Bruijn graph therefore is $|\{i \mid 1 \le i \le n-k \text{ and } S[i..i+k-1] \in V_1 \cup V_2\}|$.

## Operations on the compressed de Bruijn graph

It is our next goal to show how the combination of the implicit graph and the FM-index can be used to search for a pattern *P* of length $m\ge k$. This is important, for example, if one wants to search for a certain allele in the pan-genome and—if it is present—to examine the neighborhood of that allele in the graph. Algorithm 4 shows pseudo-code for such a search. The main difficulty is to find the node of the *k*-length suffix of *P* in the implicit graph. Once we have found this node, we can use the method introduced in the previous section to continue the search (where backward search replaces the $\mathsf {LF}$-mapping).

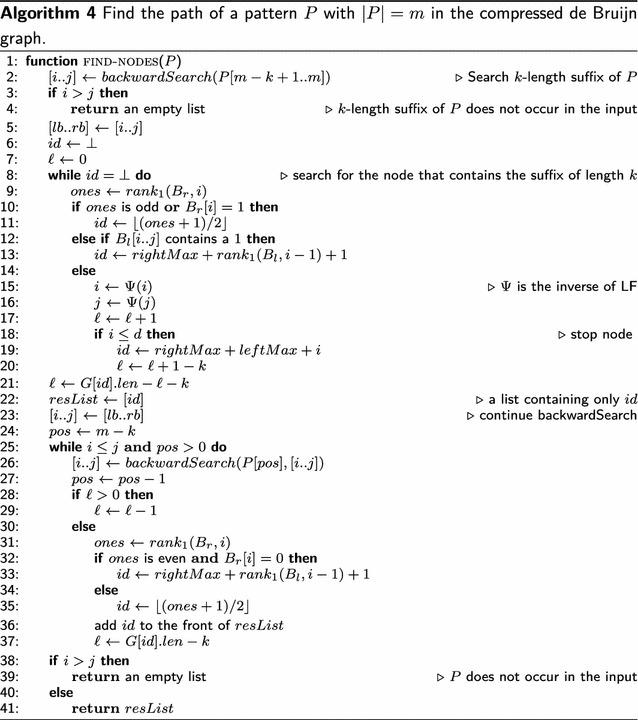



**Fig. 5:**
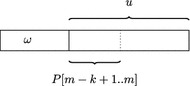
The string $\omega$ has *u* as suffix and *u* has $P[m-k+1..m]$ as prefix

Using the FM-index, we first find the suffix array interval [*i*..*j*] of the *k*-mer suffix $P[m-k+1..m]$ of *P*. If $i\le j$ (i.e., $P[m-k+1..m]$ occurs in the pan-genome), we search for the node *G*[*id*] whose corresponding string $\omega$ contains $P[m-k+1..m]$. If $P[m-k+1..m]$ is a suffix of $\omega$, then the unknown identifier *id* can be determined by lines 9–13 in Algorithm 4. If it is not a suffix of $\omega$, then there is a suffix *u* of $\omega$ that has $P[m-k+1..m]$ as prefix; see Fig. 5. The key observation is that [*i*..*j*] is the suffix array interval of *u*. Moreover, *u* can be written as $c_1c_2\dots c_{\ell } x$, where $c_q \in \Sigma$ for $q\in \{1,\dots ,\ell \}$ and *x* is the *k*-mer suffix of *u*. Note that the value of $\ell$ is unknown. Since $c_2\dots c_{\ell } x$ is not left-maximal, it follows that $[\Psi (i)..\Psi (j)]$ is its suffix array interval (this can be proven by similar arguments as in the proof of Lemma 4). Algorithm 4 iterates this process until either on line 18 the identifier of a stop node or on lines 9–13 the identifier of a non-stop-node is found. In the latter case, there are $\ell$ characters before the *k*-mer suffix *x* of *u*; so $|u|=\ell +k$ and therefore $G[id].len - \ell -k$ characters precede *u* in $\omega$ (see line 21). In the former case, $u=c_1c_2\dots c_{\ell } \#$ has length $\ell +1$ and thus $G[id].len - \ell -1$ characters precede *u* in $\omega$. To obtain this value on line 21, *k* is subtracted from $\ell +1$ on line 20.

To summarize, after $\ell$ is set to its new value on line 21 of Algorithm 4, we know that *id* is the identifier of the node whose corresponding string $\omega$ contains $P[m-k+1..m]$ and that there are $\ell$ characters preceding $P[m-k+1..m]$ in $\omega$. On line 22 the list *resList*, which will eventually contain the nodes corresponding to pattern *P*, is initialized with the element *id*. In the while-loop on lines 25–37, the backward search continues with the character *P*[*pos*] (where $pos=m-k$) and the $P[m-k+1..m]$-interval [*i*..*j*]. As long as $i\le j$ (i.e., the suffix $P[pos+1..m]$ occurs in the pan-genome) and $pos > 0$, *backwardSearch*(*P*[*pos*], [*i*..*j*]) yields the suffix array interval of *P*[*pos*..*m*] and *pos* is decremented by one. Within the while-loop there is a case distinction:

1. If $\ell > 0$, then the current prefix of *P*[*pos*..*m*] still belongs to the current node. In this case $\ell$ is decremented by one.
2. If $\ell = 0$, then the *k*-mer prefix of *P*[*pos*..*m*] belongs to the predecessor node of the current node. Its identifier *id* is determined in the usual way and then added to the front of *resList*. The variable $\ell$ is set to the new value $G[id].len-k$ because so many characters precede the *k*-mer prefix of *P*[*pos*..*m*] in the string corresponding to node *G*[*id*].

Algorithm 4 has a worst-case time complexity of $O((m+\ell ) \log \sigma )$, where $m = |P|$ and $\ell$ is the number of executions of the else-statement on line 14. This is because the overall number of backward search steps (each of which takes $O(\log \sigma )$ time) is *m* and the number of computations of $\Psi$-values (each of which also takes $O(\log \sigma )$ time) is $2\ell$. Of course, $\ell$ is bounded by the length of the longest string corresponding to a node, but this can be proportional to *n*. As a matter of fact, the worst case occurs when the algorithm gets a de Bruijn sequence of order *k* on the alphabet $\Sigma$ as input: this is a cyclic string of length $n=\sigma ^k$ containing every length *k* string over $\Sigma$ exactly once as a substring. For example, the string *aacagatccgctggtt* is a de Bruijn sequence of order $k=2$ on the alphabet $\Sigma = \{a,c,g,t\}$. The compressed de Bruijn graph for such a sequence has just one node and the corresponding string is the de Bruijn sequence itself. In practice, however, $\ell$ is rather small; see end of “Experimental results” section.

Algorithm 4 finds the nodes in the compressed de Bruijn graph that correspond to a pattern *P*. In this context, the following (and similar) questions arise:

- In which sequences (or genomes) does pattern *P* (or node *v*) occur?
- In how many sequences (or genomes) does pattern *P* (or node *v*) occur?
- How often does pattern *P* (or node *v*) occur in a specific sequence (or genome)?

To answer these questions efficiently, we employ the document array *D* of size $n=|S|$. An entry $D[i] =j$ means that the suffix $S_{\mathsf {SA}[i]}$ belongs to (or starts within) the sequence $S^j$, where $j\in \{1,\dots ,d\}$. The document array can be constructed in linear time from the suffix array or the $\mathsf {BWT}$; see e.g. [27, p. 347]. If we store the document array in a wavelet tree, then the above-mentioned questions can be answered as follows: Given the suffix array interval [*lb*..*rb*] of pattern *P* (or node *v*), the procedure call *getIntervals*([*lb*..*rb*]) on the wavelet tree of the document array returns a list consisting of all sequence numbers *j* in which *P* occurs plus the number of occurrences of *P* in $S^j$. The worst-case time complexity of the procedure *getIntervals* is $O(z + z \log (d/z))$, where *z* is the number of elements in the output list; see "Preliminaries" section.

## Experimental results

The experiments were conducted on a 64 bit Ubuntu 14.04.1 LTS (Kernel 3.13) system equipped with two ten-core Intel Xeon processors E5-2680v2 with 2.8 GHz and 128GB of RAM (but no parallelism was used). All programs were compiled with g++ (version 4.8.2) using the provided makefile. As test files we used the *E. coli* genomes listed in the supplementary material of [5]. Additionally, we used 5 different assemblies of the human reference genome (UCSC Genome Browser assembly IDs: hg16, hg17, hg18, hg19, and hg38) as well as the maternal and paternal haplotype of individual NA12878 (Utah female) of the 1000 Genomes Project; see [29]. Our software and test data are available at https://www.uni-ulm.de/in/theo/research/seqana.html; splitMEM can be obtained from http://www.sourceforge.net/projects/splitmem/.

We implemented the three algorithms $\textsf {A1}$–$\textsf {A3}$ described in the preliminary version of this article [7] and our new algorithm $\textsf {A4}$ using Simon Gog’s library sdsl [30]. Both $\textsf {A1}$ and $\textsf {A2}$ require at least $n \log n$ bits because the suffix array must be kept in main memory. Hence Yuta Mori’s fast algorithm divsufsort can be used to construct the suffix array without increasing the memory requirements. By contrast, $\textsf {A3}$ and $\textsf {A4}$ use a variant of the semi-external algorithm described in [21] to construct the $\mathsf {BWT}$. Both $\textsf {A3}$ and $\textsf {A4}$ store the $\mathsf {BWT}$ in a wavelet tree and use additional bit vectors; see “Computation of right-maximal k-mers and node identifiers” section. The variants of the algorithms that appear in Table 2 are as follows: $\textsf {A3compr1}$ and $\textsf {A4compr1}$ compress only the additional bit vectors, $\textsf {A3compr2}$ and $\textsf {A4compr2}$ also compress the (bit vectors in the) wavelet tree, whereas $\textsf {A3}$ and $\textsf {A4}$ do not use these compression options at all. In contrast to the other algorithms, $\textsf {A4}$ (and its variants) constructs the implicit graph (instead of the explicit graph) and the wavelet tree of the document array. For a comparison with the other algorithms, we also measured (called $\textsf {A4+explicit}$) the construction of the implicit and the explicit graph (i.e., the combination of Algorithms 2 and 3).

**Table 2:** Runtime and maximum main memory usage for the construction of the compressed de Bruijn graph

*k*	Algorithm	40 *E. coli*	62 *E. coli*	7 × Chr1	7 × HG
init	SplitMEM	117 (315.25)	141 (317.00)	−	−
init	A1, A2	38 (5.00)	64 (5.00)	380 (5.00)	−
init	A3, A4	131 (1.32)	202 (1.24)	1168 (1.24)	20,341 (1.24)
50	SplitMEM	2261 (572.19)	−	−	−
50	A1	57 (5.22)	92 (5.34)	596 (6.20)	−
50	A2	61 (8.49)	97 (8.78)	619 (9.98)	−
50	A3	188 (2.23)	300 (2.26)	1733 (3.07)	29,816 (2.77)
50	A3compr1	208 (1.81)	346 (1.85)	1880 (2.66)	31,472 (2.36)
50	A3compr2	236 (1.63)	374 (1.66)	2318 (2.51)	39,366 (2.22)
50	A4	164 (1.75)	254 (1.82)	1419 (1.28)	25,574 (1.96)
50	A4compr1	167 (1.46)	257 (1.53)	1435 (1.28)	25,866 (1.66)
50	A4compr2	179 (1.32)	272 (1.24)	1526 (1.24)	27,365 (1.39)
50	A4+explicit	172 (3.26)	268 (3.35)	1515 (3.59)	27,619 (3.88)
50	A4compr1+explicit	176 (2.97)	271 (3.06)	1541 (3.31)	28,044 (3.64)
50	A4compr2+explicit	188 (2.66)	289 (2.74)	1629 (2.96)	29,517 (3.38)
100	SplitMEM	2568 (572.20)	−	−	−
100	A1	59 (5.00)	95 (5.00)	595 (5.95)	−
100	A2	62 (7.89)	99 (8.19)	605 (9.74)	−
100	A3	188 (1.63)	299 (1.68)	1738 (2.74)	27,815 (2.23)
100	A3compr1	205 (1.50)	326 (1.49)	1839 (2.33)	30,401 (1.80)
100	A3compr2	232 (1.32)	411 (1.29)	2340 (2.14)	38,134 (1.66)
100	A4	174 (1.71)	261 (1.79)	1422 (1.28)	25,723 (1.94)
100	A4compr1	171 (1.42)	264 (1.50)	1439 (1.28)	26,040 (1.64)
100	A4compr2	185 (1.32)	289 (1.24)	1544 (1.24)	27,464 (1.37)
100	A4+explicit	178 (2.61)	270 (2.73)	1486 (3.21)	26,878 (3.36)
100	A4compr1+explicit	175 (2.32)	273 (2.44)	1500 (2.92)	26,999 (3.07)
100	A4compr2+explicit	190 (2.01)	299 (2.12)	1624 (2.68)	28,665 (2.80)
500	SplitMEM	2116 (570.84)	−	−	−
500	A1	72 (5.00)	113 (5.00)	620 (5.83)	−
500	A2	83 (7.17)	117 (7.43)	640 (9.66)	−
500	A3	194 (1.50)	304 (1.49)	1752 (2.67)	28,548 (2.07)
500	A3compr1	216 (1.50)	325 (1.49)	1839 (2.19)	30,488 (1.65)
500	A3compr2	241 (1.32)	378 (1.29)	2319 (2.06)	36,993 (1.50)
500	A4	184 (1.65)	283 (1.74)	1453 (1.28)	26,362 (1.93)
500	A4compr1	197 (1.35)	287 (1.44)	1477 (1.28)	26,545 (1.63)
500	A4compr2	213 (1.32)	322 (1.24)	1622 (1.24)	28,501 (1.36)
500	A4+explicit	185 (1.81)	285 (1.90)	1509 (3.14)	27,285 (3.14)
500	A4compr1+explicit	198 (1.52)	288 (1.61)	1535 (2.83)	27,417 (2.79)
500	A4compr2+explicit	214 (1.32)	323 (1.29)	1694 (2.56)	29,283 (2.58)

The first column shows the *k*-mer size (an entry init means that only the index data structure is constructed) and the second column specifies the algorithm used in the experiment. The remaining columns show the run-times in seconds and, in parentheses, the maximum main memory usage in bytes per base pair (including the construction) for the data sets described in the text. A minus indicates that the respective algorithm was not able to solve its task on our machine equipped with 128 GB of RAM

The first part of Table 2 (in which the *k* column has the entries init) shows how much time (in seconds) an algorithm needs to construct the index data structure and its maximum main memory usage in bytes per base pair. In the experiments, we built compressed de Bruijn graphs for the 62 *E. coli* genomes (containing 310 million base pairs) using the *k*-mer lengths 50, 100, and 500. Table 2 shows the results of these experiments. The run-times include the construction of the index, but similar to splitMEM it is unnecessary to rebuild the index for a fixed data set and varying values of *k*. The peak memory usage reported in Table 2 includes the size of the index *and* the size of the compressed de Bruijn graph. Due to its large memory requirements, splitMEM was not able to build a compressed de Bruijn graph for all 62 strains of *E. coli* on our machine equipped with 128 GB of RAM. That is why we included a comparison based on the first 40 *E. coli* genomes (containing 199 million base pairs) of the data set. The experimental results show that our algorithms are more than an order of magnitude faster than splitMEM while using significantly less space (two orders of magnitude). To show the scalability of the new algorithms, we applied them to different assemblies of the human genome (consisting of 23 chromosomes: the 22 autosomes and the X-chromosome). The compressed de Bruijn graphs of their first chromosomes (7 × Chr1, containing 1736 million base pairs) and the complete seven genomes (7 × HG, containing 21,201 million base pairs) were built for the *k*-mer lengths 50, 100, and 500. One can see from Table 2 that algorithms $\textsf {A1}$ and $\textsf {A2}$ are very fast, but 128 GB of RAM was not enough for them to successfully build the graph for the seven human genomes (note that at least 5 bytes per base pair are required). So let us compare algorithms $\textsf {A3}$ and $\textsf {A4}$ (and their variants). The construction of the explicit graph with $\textsf {A4+explicit}$ is faster than with $\textsf {A3}$, but $\textsf {A4+explicit}$ seems to use much more space for this task. The space comparison, however, is not fair because $\textsf {A4}$ also constructs the wavelet tree of the document array and two select data structures for the wavelet tree of the $\mathsf {BWT}$ to calculate $\Psi$ values. These data structures are important for searches on the graph, but they are superfluous in the construction of the explicit graph. So in fact $\textsf {A4+explicit}$ uses only a little more space for this task because the implicit representation of the graph, which must be kept in main memory, is rather small. Table 4 contains a detailed breakdown of the space usage of the variants of algorithm $\textsf {A4}$.

**Table 3:** Space in bytes per input base pair for the explicit and the implicit representation of the compressed de Bruijn graph

k	ds	40 *E. coli*	62 *E. coli*	7 × Chr1	7 × HG
50	Explicit	1.80	1.89	2.80	2.57
50	Implicit	0.84	0.82	0.77	0.76
50	Implicit-c1	0.55	0.53	0.47	0.47
50	Implicit-c2	0.30	0.27	0.25	0.26
100	Explicit	1.46	1.51	2.55	2.12
100	Implicit	0.80	0.79	0.75	0.74
100	Implicit-c1	0.51	0.50	0.46	0.45
100	Implicit-c2	0.26	0.24	0.23	0.24
500	Explicit	1.07	1.08	2.50	2.01
500	Implicit	0.74	0.74	0.75	0.74
500	Implicit-c1	0.44	0.44	0.45	0.44
500	Implicit-c2	0.20	0.18	0.23	0.23

The numbers for the explicit representation include the input and the numbers for the implicit representation include the $\mathsf {BWT}$ stored in a wavelet tree. The suffix -c1 means that the bit vectors $B_{l}$ and $B_{r}$ of the implicit representation are compressed, and the suffix -c2 means that additionally the (bit vectors in the) wavelet tree are compressed

As the explicit compressed de Bruijn graph, the combination of the implicit graph and the FM-index supports a graph traversal (albeit in backward direction). For this task the implicit graph and the FM-index use much less space than the explicit graph. This can be seen as follows. One can store the explicit representation by using—for each node—an integer for *len* and a pointer to *posList*/*adjList*. Additionally, each edge causes an entry in the *posList* and an entry in the *adjList* of a node. Altogether, this requires two integers per node and two integers per edge. In contrast, the implicit representation needs four integers per node $(len,lb,size,{ suffix}\_lb)$ and (independent of the number of edges) the two bit vectors $B_{l}$ and $B_{r}$, both equipped with an additional data structure that supports rank queries in O(1) time. Table 3 shows the measured space usage of the different representations of the compressed de Bruijn graph.

In contrast to the explicit graph, our new data structure allows to search for a pattern *P* in the graph and to answer questions like: In how many sequences does *P* occur? It is this new functionality (notably the document array) that increases the memory usage again; cf. Table 4. Despite this new functionality, the overall space consumption of $\textsf {A4}$ is in most cases less than that of $\textsf {A3}$; see Table 2.

**Table 4:** Breakdown of the space usage of the variants of algorithm A4

Algo	Part	62 *E. coli*	7 × Chr1	7 × HG
A4	Wt-bwt	0.42 (23.83 %)	0.44 (36.23 %)	0.43 (22.68 %)
A4	Nodes	0.10 (5.94 %)	0.03 (2.61 %)	0.04 (2.02 %)
A4	$B_{r}$	0.16 (8.93 %)	0.16 (12.86 %)	0.16 (8.25 %)
A4	$B_{l}$	0.14 (8.04 %)	0.14 (11.57 %)	0.14 (7.42 %)
A4	Wt-doc	0.93 (53.26 %)	0.45 (36.73 %)	1.13 (59.63 %)
A4compr1	Wt-bwt	0.42 (28.57 %)	0.44 (47.83 %)	0.43 (26.85 %)
A4compr1	Nodes	0.10 (7.12 %)	0.03 (3.44 %)	0.04 (2.39 %)
A4compr1	$B_{r}$	0.00 (0.23 %)	0.00 (0.12 %)	0.00 (0.09 %)
A4compr1	$B_{l}$	0.00 (0.23 %)	0.00 (0.12 %)	0.00 (0.08 %)
A4compr1	Wt-doc	0.93 (63.85 %)	0.45 (48.49 %)	1.13 (70.59 %)
A4compr2	Wt-bwt	0.16 (13.03 %)	0.22 (31.01 %)	0.22 (15.62 %)
A4compr2	Nodes	0.10 (8.67 %)	0.03 (4.55 %)	0.04 (2.76 %)
A4compr2	$B_{r}$	0.00 (0.28 %)	0.00 (0.16 %)	0.00 (0.10 %)
A4compr2	$B_{l}$	0.00 (0.28 %)	0.00 (0.16 %)	0.00 (0.10 %)
A4compr2	Wt-doc	0.93 (77.74 %)	0.45 (64.11 %)	1.13 (81.42 %)

The first column shows the algorithm used in the experiment (the *k*-mer size is 50). The second column specifies the different data structures used: wt-bwt stands for the wavelet tree of the $\mathsf {BWT}$ (including rank and select support), nodes stands for the array of nodes (the implicit graph representation), $BV_r$ and $BV_l$ are the bit vectors described in "Computation of right-maximal k-mers" section (including rank support), and wt-doc stands for the wavelet tree of the document array. The remaining columns show the memory usage in bytes per base pair and, in parentheses, their percentage

In our next experiment, we measured how long it takes to find the nodes in the graph that correspond to a pattern *P*. Since the median protein length in *E. coli* is 278 and a single amino acid is coded by three nucleotides, we decided to use a pattern length of 900. Table 5 shows the results for 10,000 patterns that occur in the pan-genome (if patterns do not occur in the pan-genome, the search will be even faster; data not shown). Furthermore, we measured how long it takes to determine to which sequences each node belongs (using the procedure *getIntervals* on the wavelet tree of the document array as described at the end of "Operations on the compressed de Bruijn graph" section). Table 6 shows the results for the nodes corresponding to 10,000 patterns that occur in the pan-genome.

**Table 5:** Runtime and main memory usage for finding nodes

*k*		62 *E. coli*	7 × Chr1	7 × HG
50	A4	3 (1.81)	9 (1.28)	9 (1.96)
50	A4compr1	3 (1.52)	9 (0.98)	11 (1.66)
50	A4compr2	6 (1.20)	20 (0.70)	29 (1.39)
100	A4	3 (1.78)	12 (1.26)	27 (1.94)
100	A4compr1	3 (1.49)	15 (0.97)	19 (1.64)
100	A4compr2	6 (1.17)	31 (0.68)	51 (1.37)
500	A4	9 (1.73)	20 (1.26)	22 (1.93)
500	A4compr1	12 (1.43)	24 (0.96)	27 (1.63)
500	A4compr2	17 (1.11)	55 (0.67)	74 (1.36)

The first column shows the *k*-mer size and the second column specifies the algorithm used in the experiment. The remaining columns show the run-times in seconds for finding the nodes corresponding to 10,000 patterns of length 900 (that occur in the pan-genome) and, in parentheses, the maximum main memory usage in bytes per base pair for the data sets described in the text

**Table 6:** Runtime and main memory usage for finding sequences that correspond to given nodes

*k*		62 *E. coli*	7 × Chr1	7 × HG
50	A4	10.84 (1.81)	3.31 (1.28)	15.33 (1.96)
50	A4compr1	10.91 (1.52)	3.17 (0.98)	14.88 (1.66)
50	A4compr2	11.02 (1.20)	3.07 (0.70)	13.02 (1.39)
100	A4	8.31 (1.78)	2.72 (1.26)	10.99 (1.94)
100	A4compr1	8.11 (1.49)	2.83 (0.97)	9.10 (1.64)
100	A4compr2	8.23 (1.17)	2.84 (0.68)	9.25 (1.37)
500	A4	2.43 (1.73)	1.32 (1.26)	4.51 (1.93)
500	A4compr1	2.78 (1.43)	1.32 (0.96)	4.22 (1.63)
500	A4compr2	2.32 (1.11)	1.29 (0.67)	4.30 (1.36)

The first column shows the *k*-mer size and the second column specifies the algorithm used in the experiment. The remaining columns show the run-times in seconds for finding out to which sequences each of the nodes belongs (where the nodes correspond to 10,000 patterns of length 900 that occur in the pan-genome) and, in parentheses, the maximum main memory usage in bytes per base pair for the data sets described in the text

Finally, we determined the length of the longest string corresponding to a node in the compressed de Bruijn graph. This is important because the worst-case search time depends on this length; see end of "Construction of the explicit compressed de Bruijn graph" section. The results can be found in Table 7.

**Table 7:** Length of the longest string corresponding to a node

*k*	62 *E. coli*	7 x Chr1	7 x HG
50	79,967	41,571	36,579
100	173,366	85,773	203,398
500	179,671	2,283,980	1,402,896

The first column specifies the *k*-mer size and the remaining columns show the length of the longest string corresponding to a node in the compressed de Bruijn graph

## Conclusions

We have presented a space-efficient method to build the compressed de Bruijn graph from scratch. An experimental comparison with splitMEM showed that our algorithm is more than an order of magnitude faster than splitMEM while using significantly less space (two orders of magnitude). To demonstrate its scalability, we successfully applied it to seven complete human genomes. Consequently, it is now possible to use the compressed de Bruijn graph for much larger pan-genomes than before (consisting e.g. of hundreds or even thousands of different strains of bacteria). Moreover, the combination of the implicit graph and the FM-index can be used to search for a pattern *P* in the graph (and to traverse the graph).

Future work includes a parallel implementation of the construction algorithm. Moreover, it should be worthwhile to investigate the time-space trade-off if one uses data structures that are optimized for highly repetitive texts; see [31] and the references therein.
