# Erratum to: A representation of a compressed de Bruijn graph for pan-genome analysis that enables search

**DOI:** 10.1186/s13015-016-0090-8

**Published:** 2016-11-28

**Authors:** Timo Beller, Enno Ohlebusch

**Affiliations:** Institute of Theoretical Computer Science, Ulm University, James-Franck-Ring O27/537, 89069 Ulm, Germany

## Erratum to: Algorithms Mol Biol (2016) 11:20 DOI 10.1186/s13015-016-0083-7

After publication of the original article [1], the authors noticed errors in Algorithm 2 and the caption of Table 4. In Algorithm 2, the term “rank_1_(B_l_, i − 1) + 1” should be included on line 28 and not line 29. In addition, in the caption of Table 4, the word “BV_r_” should be replaced by “B_r_” and the word “BV_l_” should be replaced by “B_l_”. The correct versions of Algorithm 2 and Table 4 are included in this erratum.
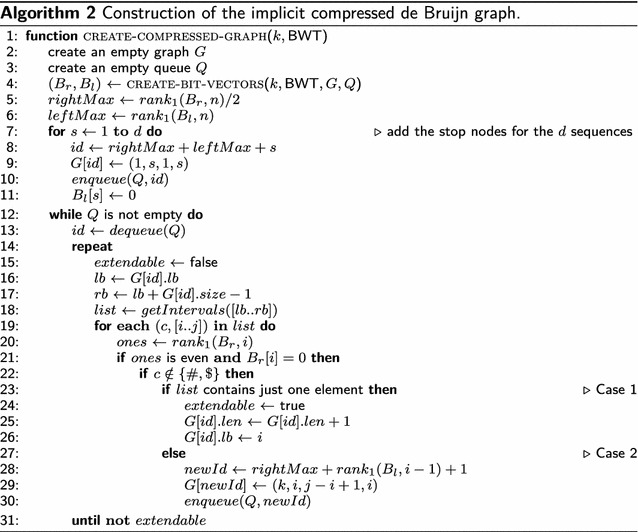


**Table 4:** Breakdown of the space usage of the variants of Algorithm A4

Algorithm	Part	62 *E.coli*	7 × Chr1	7 × HG
A4	wt-bwt	0.42 (23.83%)	0.44 (36.23%)	0.43 (22.68%)
A4	Nodes	0.10 (5.94%)	0.03 (2.61%)	0.04 (2.02%)
A4	$B_r$	0.16 (8.93%)	0.16 (12.86%)	0.16 (8.25%)
A4	$B_l$	0.14 (8.04%)	0.14 (11.57%)	0.14 (7.42%)
A4	wt-doc	0.93 (53.26%)	0.45 (36.73%)	1.13 (59.63%)
A4compr1	wt-bwt	0.42 (28.57%)	0.44 (47.83%)	0.43 (26.85%)
A4compr1	Nodes	0.10 (7.12%)	0.03 (3.44%)	0.04 (2.39%)
A4compr1	$B_r$	0.00 (0.23%)	0.00 (0.12%)	0.00 (0.09%)
A4compr1	$B_l$	0.00 (0.23%)	0.00 (0.12%)	0.00 (0.08%)
A4compr1	wt-doc	0.93 (63.85%)	0.45 (48.49%)	1.13 (70.59%)
A4compr2	wt-bwt	0.16 (13.03%)	0.22 (31.01%)	0.22 (15.62%)
A4compr2	Nodes	0.10 (8.67%)	0.03 (4.55%)	0.04 (2.76%)
A4compr2	$B_r$	0.00 (0.28%)	0.00 (0.16%)	0.00 (0.10%)
A4compr2	$B_l$	0.00 (0.28%)	0.00 (0.16%)	0.00 (0.10%)
A4compr2	wt-doc	0.93 (77.74%)	0.45 (64.11%)	1.13 (81.42%)

The first column shows the algorithm used in the experiment (the *k*-mer size is 50). The second column specifies the different data structures used: wt-bwt stands for the wavelet tree of the $\mathsf {BWT}$ (including rank and select support), nodes stands for the array of nodes (the implicit graph representation), $B_r$ and $B_l$ are the bit vectors described in “Computation of right-maximal *k*-mers and node identifiers” section (including rank support), and wt-doc stands for the wavelet tree of the document array. The remaining columns show the memory usage in bytes per base pair and, in parentheses, their percentage
